# Understanding the Antifungal Mechanism of Ag@ZnO Core-shell Nanocomposites against *Candida krusei*

**DOI:** 10.1038/srep36403

**Published:** 2016-11-04

**Authors:** Bhaskar Das, Md. Imran Khan, R. Jayabalan, Susanta K. Behera, Soon-Il Yun, Suraj K. Tripathy, Amrita Mishra

**Affiliations:** 1 School of Biotechnology, KIIT University, Bhubaneswar 751024, India; 2 Department of Life Sciences, National Institute of Technology, Rourkela 6150, India; 3 IMGENEX India Pvt. Ltd., Bhubaneswar 751024, India; 4 Department of Food Science & Technology, Chonbuk National University, Jeonju 561756, South Korea; 5 School of Applied Sciences, KIIT University, Bhubaneswar 751024, India

## Abstract

In the present paper, facile synthesis of Ag@ZnO core-shell nanocomposites is reported where zinc oxide is coated on biogenic silver nanoparticles synthesized using *Andrographis paniculata* and *Aloe vera* leaf extract. Structural features of as synthesized nanocomposites are characterized by UV-visible spectroscopy, XRD, and FTIR. Morphology of the above core-shell nanocomposites is investigated by electron microscopy. As synthesized nanocomposite material has shown antimicrobial activity against *Candida krusei*, which is an opportunistic pathogen known to cause candidemia. The possible mode of activity of the above material has been studied by *in-vitro* molecular techniques. Our investigations have shown that surface coating of biogenic silver nanoparticles by zinc oxide has increased its antimicrobial efficiency against *Candida krusei*, while decreasing its toxicity towards A431 human epidermoid carcinoma cell lines.

Fungal infections caused by *Candida* species have become one of the major causes of morbidity and mortality in immunocompromised situation, particularly for patients with hematologic malignancies and for transplant recipients^1^,^2^,^3^,^4^. Despite sincere efforts, treatment of candidemia remains a challenging issue due to frequent development of resistance to the antifungal drugs used in clinical settings^5^. For many years, most of the cases of candidemia were caused by *Candida albicans*. Recently, other *Candida* species have proved to be responsible for an increased proportion of candidemia. In particular, *Candida krusei* (*C. krusei*) has become one of the most frequent of these emerging pathogens since it is opportunistic in nature showing resistance to commonly used drugs^6^,^7^. It is most often found in patients who have had prior fluconazole exposure^8^. More recent studies have shown infections due to *C. krusei* among patients receiving amphotericin B^9^. While great effort is made to understand the biology, epidemiology and pathogenicity of this pathogen, little success has been achieved in developing new treatment strategies^10^. Thus, there is an urgent need to search for alternative antifungal agents. In this regard, nanomaterials based strategies have received much attention by the scientific community owing to their unique antimicrobial effectiveness caused by combination of their small size and high surface-to-volume ratio, which enable intimate interactions with microbial membranes^11^,^12^. Inorganic antibacterial agents such as metallic and metal oxide nanoparticles (NPs) are considered to be advantageous compared to organic compound due to their stability, chemical inertness, and potential biotechnological applications^13^,^14^. Out of various nano-structured materials under investigation, Ag NPs have shown interesting antifungal activity against *Candida albicans*. Since, there are several reports on acute toxic effects of Ag NPs against human cell lines; its practical application is hindered^15^,^16^. To overcome these limitations, researchers have been trying to develop nanocomposites (NC) which may preserve the antibacterial activity of the metallic NPs while increasing the biocompatibility of the resultant composite structure. NC materials containing metallic silver has been explored as antibacterial agents for a long time. Ag/SiO_2_ NC are studied for their potential antibacterial effect against *Escherichia coli* and *Staphylococcus aureus*^17^,^18^,^19^,^20^. Zhang and co-workers have reported the antibacterial effect of Ag/TiO_2_ NC powders against *Escherichia coli*^21^. Sao *et al*. have synthesized Ag/graphene oxide NC with remarkable activity against *Escherichia coli* and *S. aureus*^22^. Although these materials have demonstrated potential antibacterial behavior, their clinical trial has been delayed due to possible toxic behavior towards healthy cells and tissues^15^,^16^. A major goal of designing NC is to tailor the antimicrobial properties of the resultant system by manipulating their chemical composition and/or morphologies while offering little or no collateral damage to the healthy cells and/or tissues. Although the metal/metal oxide based NC could show interesting antimicrobial activity, they have never been tried against fungal species. One of the major challenges in using NC systems for fungal species is their possible toxic impact on the host at an enhanced dose size. To deal with these disadvantages, scientists have been trying to develop NC with core-shell morphology, which may decrease the toxic effect of the metal NPs. Core-shell morphology can have advantages over traditional NC systems not only because they will protect the metal NPs against agglomeration and leaching out in real working environment but also may maintain the antifungal activity of the metallic core^18^,^23^. However, to the best of our knowledge any core-shell NC system has not been investigated for antifungal application.

Keeping this in view; present study reports the synthesis of Ag@ZnO core-shell NC, where Ag NPs are coated with thin layer of ZnO. ZnO is known to be less toxic than metallic NPs and hence it is expected that formation of oxide layer will decrease the toxicity of silver. Additionally, earlier studies by He *et al*. and Gondal *et al*. have shown significant antifungal behavior of ZnO against *Botrytis cinerea*, *Penicillium expansum* and *Candida albicans*^24^,^25^. Thus it is proposed that the resultant NC may have similar activity against the target pathogen i.e. *Candida krusei*. Ag NPs were synthesized by using *Andrographis paniculata* and *Aloe vera* leaf extract followed by coating of ZnO by using zinc nitrate hexahydrate in alkaline condition. Antifungal activity of the NC system has been studied against *C. krusei*. In order to understand the possible antifungal mechanism of the synthesized NC against *C. krusei,* other studies such as lipid perooxidation, measurement of reactive oxygen species, DNA degradation by agarose gel electrophoresis was also carried out. The cytotoxicity of the Ag@ZnO NC has been compared with that of biogenic Ag NPs using A431 human epidermoid carcinoma cell lines.

## Results and Discussion

### UV-visible spectroscopy

**Figure 1:**
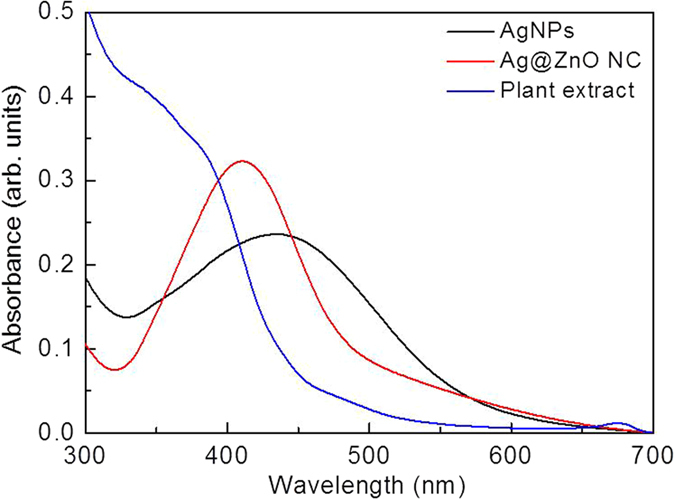
UV-spectrum of Ag NPs, Ag/ZnO, and only plant extract.

Due to presence of free surface electrons, nobel metal NPs show strong surface Plasmon resonance (SPR) band in the UV-visible spectrum. Thus, bio-synthesis of Ag NPs has been followed by change in the SPR band of the aqueous dispersion. Surface coating with a metal oxide layer is expected to change the dielectric constant of the surrounding environment and hence can shift the SPR band of metal NPs. This concept has been extensively used to monitor the formation of metal@metal oxide core-shell NC^17^,^23^. UV-visible spectra of the aqueous dispersion of Ag NPs, Ag@ZnO core-shell NC, and only plant extract are shown in Fig. 1. Aqueous dispersion of biogenic Ag NPs showed a clear SPR band at 434 nm^17^. After the addition of aqueous sodium zincate sol, SPR band has shown a distinct blue shift of about 23 nm. This could be attributed to the formation of the oxide shell on the surface of Ag NPs. It has been previously demonstrated that the SPR band is highly sensitive to a variety of perturbations on the particle surface^17^,^23^. For ZnO (*n*_*ZnO*_ = 1.92) with a refractive index remarkably higher than that of water (*n*_*H2O*_ = 1.33), strong blue-shifts in the SPR band position is expected for Ag colloidal particles as they become encapsulated with zinc oxide shell. This interesting optical phenomenon is associated with the transfer of electronic charge between the core and shell. Ag NPs have negative charge due to excess surface electrons which causes the rapid aggregation of the colloid^17^,^24^,^25^. Formation of an oxide shell helps to accommodate the excess electrons present on the metal surface. Due to the large difference between the intrinsic Fermi level of the core and the conduction band energy of the (n-type) semiconductor shell, mobile electrons that diffuse within the shell will be trapped for long periods of time in the core^17^. This reduces the possibility of interaction between the adjacent metal NPs and hence may increase the stability of the resultant NC system. In the aqueous condition, zinc nitrate is expected to undergo rapid dissolution to produce Zn^2+^ and NO^3–^ ions. In presence of sodium hydroxide Zn^2+^ may precipitate rapidly to form Zn(OH)_2_. In presence of high concentration of NaOH and water, the resultant Zn(OH)_2_ would not simply undergo a thermal decomposition to produce ZnO and H_2_O rather it is expected to produce soluble Na_2_ZnO_2_. However in aqueous solutions principal zincate ion appears as [Zn(OH)_3_.H_2_O]^−^. Now under hydrothermal condition, solvated zincate ion converts to produce [Zn(OH)_4_]^2−^. These [Zn(OH)_4_]^2−^ ions are expected to nucleate on the silver nanoparticle surface and can proceed in two different ways. It may directly undergo thermal decomposition to produce ZnO and H_2_O or it may convert to Zn(OH)_2_ which further decomposes to produce ZnO^17^.

### X-ray diffraction studies

**Figure 2:**
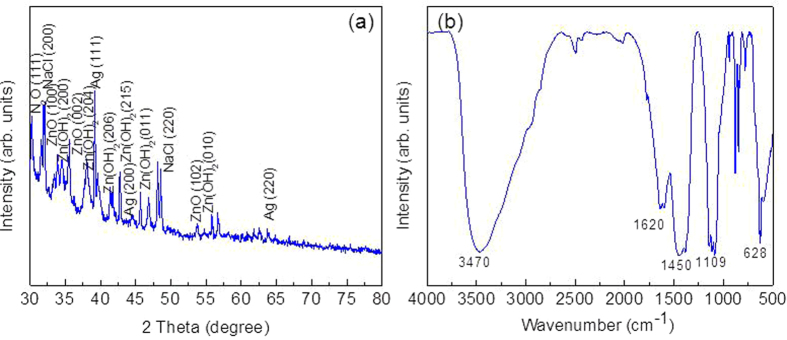
Characterization of Ag@ZnO NC (**a**) X-ray Diffraction (XRD) (**b**) FTIR.

Phase and crystal structure of the Ag@ZnO core-shell NC are investigated by XRD and result is shown in Fig. 2(a). For as synthesized NC, one distinct peak at 2θ = 38.1, 44.8 and 64.6 corresponding to (111), (200) and (220) planes of metallic silver with face-centered cubic structure (JCPDS Card No. 04–0783) is observed. Similarly three major peaks of ZnO at 2θ = 31.95, 34.5, and 46.8 corresponding to (100), (002), and (102) planes of synthetic ZnO with hexagonal wurtzite structure (JCPDS Card No. 36–1451) are obtained. In addition to these phases, six minor peaks (2θ = 33.83, 35.5, 39.1, 41.37, and 48.1) corresponding to (202), (204), (206), (215), and (011) peaks of orthogonal Zn(OH)_2_ (JCPDS Card No. 41.1359) are also noticed. Two minor peaks (2θ = 31.51 and 45.64) corresponding to (200) and (220) planes of sodium chloride and two minors peaks (2θ = 30.23 and 39.6) of N_2_O [corresponding to (111) and (112) planes] are also obtained. Any peak corresponding to other Ag/Zn compounds is not observed. This suggests that no Zn_1-x_Ag_x_O solid solution is formed. Mean crystallite diameter of is calculated using Scherrer’s equation and found to be ≈ 15 and 25 nm for Ag and ZnO NPs respectively.

### FTIR spectroscopy

The presence of adsorbed molecules and/or functional groups on surface of Ag@ZnO NC was analyzed with FTIR spectroscopy at room temperature in an acquired range of 500–4000 cm^‒1^. Figure 2(b) shows the FTIR spectra of as-synthesized Ag@ZnO core-shell NC. A broad band at 3200–3600 cm^‒1^ may have originated from polyphenols that are present in the extract. The small vibration at 1620 could be attributed to the stretching peak of C = O group^26^ present in the polyphenol (e.g. gallic acid) content of plant extract. The boarding band frequency of side chain of alkane groups (sp^3^ C–H bending, 1460 cm^‒1^) is obtained at 1450 cm^‒1 ^27,28. The weak band of C-N group is shown at 1109 cm^‒1^ which comes in the range of 1080–1360 cm^‒1 ^29.

### Total Phenolic content Detemination

Supplementary Figure 2 reveals that the phenolic content present in Ag@ZnO core-shell NCs is lesser than the phenolic content present in the extract solution. The decrement in the phenolic content (GAE (μg/ml)) of Ag@ZnO NCs in comparison with the *Andrographis paniculata* and Aloe vera plant extract alone or together indicates possible explanation for the synthesis mechanism of Ag@ZnO core shell NCs, where components with phenolic groups (equivalent to galic acid) present in the plant extract solution may have acted as a reducing and/or stabilizing agent.

### Transmission electron microscopy

**Figure 3:**
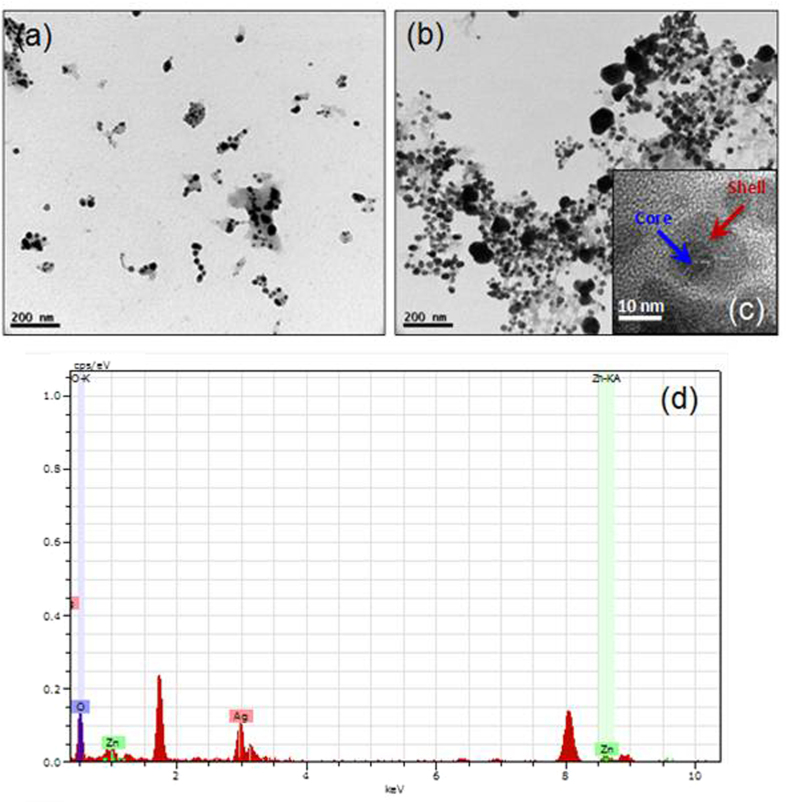
Transmission electron microscopy (TEM) images of **(a)** Ag NPs **(b)** Ag@ZnO NC, **(c)** HRTEM image and **(d)** EDAX pattern of Ag@ZnO NC.

Morphology of the Ag NPs and Ag@ZnO NC is investigated by TEM and resultant images are shown in Fig. 3. TEM samples were prepared by dipping the TEM grid in aqueous dispersion of nanomaterials followed by freeze drying for 12 h. Well-dispersed Ag NPs are clearly observed. Approximate size of the Ag NP is 30∼35 nm. ZnO NPs are found in the broad size range of 40∼75 nm. Comparison of these results with XRD data suggests the formation of polycrystalline materials. Core-shell structure is observed for Ag@ZnO NCs. However, particles are obtained with multiple silver cores encapsulated inside single zinc oxide shell. This observation was in accordance with the data obtained from UV-visible spectrum of the core-shell NCs.

### Investigation of size distribution and surface charge

Size distribution of the synthesized Ag@ZnO NCs is investigated by differential light scattering (DLS) technique. As shown in Supplementary Figure 3a NC particles were found to be in the average size range of 42 nm. The surface charge of the NC particles was investigated by zeta-potential measurement and was found to be ≈−27 mV (Supplementary Figure 3b).

### Antifungal activity against *C. krusei*

**Figure 4:**
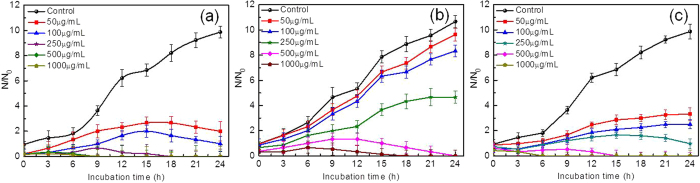
Comparative antifungal activity of NPs and NC against *Candida krusei* (**a**) Biogenic Ag NPs (**b**) chemically synthesized ZnO NPs (**c**) Ag@ZnO hybrid NC.

The comparative antifungal activity of Ag NPs, ZnO NPs and Ag@ZnO NC has been shown in Fig. 4. It can be observed that Ag NPs at a concentration of 500 and 1000 μg/mL have completely inhibited the growth of *C. krusei* following 15 h and 6 h of incubation respectively. The MIC value for biosynthesized Ag NPs is taken as 500 μg/mL. Although lower concentrations of the metal NPs (50, 100 and 250 μg/mL) have shown antifungal activity, but complete growth inhibition has not been observed. Similar results have been obtained with lower concentrations of chemically synthesized ZnO NPs. 500 μg/mL of ZnO NPs completely inhibited the growth of the organism and hence this concentration is taken as the MIC value. However the efficiency as compared to biosynthesized Ag NPs is less because the time taken to completely suppress the growth of the microbe is quite high (24 h) with ZnO NPs compared to Ag NPs. Ag@ZnO NC have shown the highest antifungal efficiency against *C. krusei*. 250, 500 and 1000 μg/mL of the NC have completely inhibited the growth of the fungus within 18, 9 and 6 h of incubation respectively. Hence 250 μg/mL of the core shell NC has been taken as the MIC value for completely killing the microbe. Lower concentrations (50 and 100 μg/mL) have suppressed the growth of the fungus by more than 50% compared to untreated cells. Earlier reports with chemically synthesized Ag-ZnO NC have shown similar results against GFP expressing antibiotic resistant *E. coli*^30^. The MIC value with their material is observed to be 550 μg/mL. Lu *et al*. have previously reported that the MIC value for *E. coli* and *S. aures* is 600 and 400 μg/mL respectively with Ag-ZnO NC system^31^. In both the studies the MIC values obtained using Ag-ZnO NC is higher as compared to the core-shell NC used in the present study against the fungus *C. krusei*.

**Figure 5:**
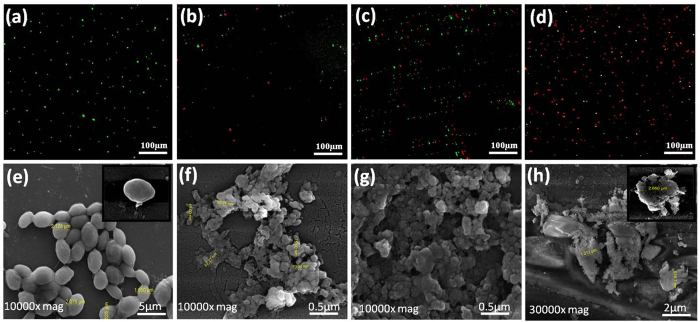
Live & dead staining of *Candida krusei* cells **(a)** Untreated **(b)** 6 h treated **(c)** 12 h treated **(d)** 24 h treated. The cells were incubated with 20 μg/mL propidium iodide (PI) and 10 μg/mL fluorescence diacetate (FDA) and examined under fluorescence microscope at 40X. FESEM of untreated and treated *Candida krusei* cells (**e**) untreated (**f**) treated cells following 6 h incubation **(g)** 12 h incubation and **(h)** 24 h incubation. For both experiments *Candida krusei* cells were incubated with 250 μg/mL of Ag@ZnO NC.

To confirm the cell viability, fluorescence based live dead staining assay has been done (Fig. 5a–d). Figure 5a represents untreated cells which are used as control experiment. From Fig. 5b, it is observed that the live fungal cells have considerably decreased in number within 6 h of treatment with 250 μg/mL of core-shell structure NC material. Following 12 h of treatment, the percentage of dead cells has considerably increased. By the end of 24 h, the live fungal count observed is almost negligible indicating very high cell death. With increase in the incubation time there is a corresponding increase in the percentage of dead cells. This might be due to the fact that increased incubation time favored more interaction of the fungal cells with the as synthesized NC material. Hence the cell death has occurred in time dependant manner. FE-SEM images of the untreated and treated cells have been shown in Fig. 5e–h respectively. From Fig. 5e, we can observe that the cells of *C. krusei* are intact having smooth surface. However in treated cells (Fig. 5f), within 6 h of treatment partial change in cell morphology has been noticed. 12 h of incubation has indicated clustering of the cells with breaks in the cell membranes (Fig. 5g). Formation of hollow pits has also been observed in the images. Cell shrinkage has been observed within 24 h of incubation with core-shell NC material (Fig. 5h). This might be due to damage of the cell membrane resulting in leakage of intracellular compounds leading to complete cell disruption. There are similar reports with ZnO NPs, Ag NPs and chemically synthesized Ag-ZnO NC against different microbes^32^,^33^. Interestingly, as compared to other *Candida* sps, *C. krusei* has multilayered cell wall and trilaminar cell membrane and hence it is very difficult to penetrate the cell by damaging the outer protective layer^34^. In the present study the effectiveness of Ag@ZnO NC against this microbe is very high which could be useful for future application purpose. Moreover there is hardly any report available on the mode of antifungal action of nanomaterial based core-shell structures against this pathogen.

### Antifungal mechanism

**Figure 6:**
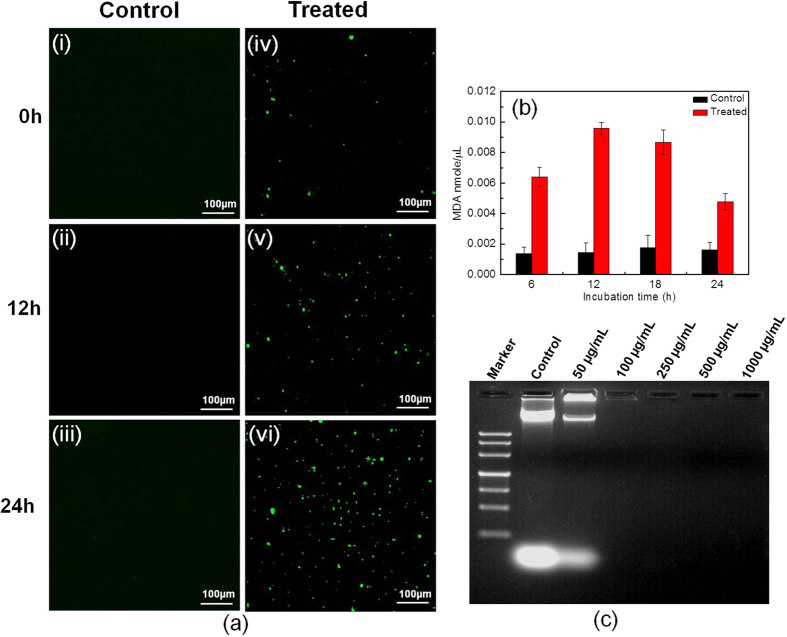
Analysis of antifungal mechanism of Ag@ZnO NC. (**a**) Generation of ROS **(i-iii)** Untreated **(iv-vi)** treated with 250 μg/mL Ag@ZnO NC (**b**) Lipid peroxidarion **(c)** DNA Degradation.

Formation of ROS in microorganisms occurs due to metabolic activity of the cells. Particularly the accumulation of ROS plays an important role in inducing apoptosis in metazoans and yeasts^35^. Therefore determination of intracellular ROS plays a significant role in underlying the probable antimicrobial mechanism of a particular compound. Figure 6a shows the generation of intracellular ROS induced by NC material against *C. krusei*. Untreated cells [Fig. 6a(i–iii)] have not shown any production of ROS with respect to increase in incubation time from 0–24 h. However treated cells [Fig. 6a(iv–vi)] have shown an increase in generation of ROS in a time dependant manner. Production of ROS has increased two to three folds within 24 h of incubation. Similarly we have studied the production of ROS with Ag and ZnO NPs. The obtained data has shown the generation of ROS in both the synthesized NPs. However, ROS generation is singificantly higher cells treated with Ag NPs compared to ZnO NPs (Supplementary Figure 4). However, the highest level of ROS has been generated with Ag@ZnO NC. We have qualitatively determined the ROS generation by using DCF-DA which can easily detect intracellular hydrogen peroxide in intact cells. DCF-DA is permeable to plasma membrane and is hydrolyzed into the cytoplasm to form DCFH- carboxylate anion^36^,^37^. From this observation, we may expect that the antimicrobial mechanism of as synthesized Ag NPs, ZnO NPs and Ag@ZnO NC against the tested pathogen is due to generation of ROS. It is well known that ROS targets biological cellular compounds and particularly lipid molecules which are highly complex in nature. In the present study we have tried to determine the effect of all the three nanomaterials on the cell membrane of the targeted pathogen by measuring MDA concentration which is one of the most important degradation products of lipid perooxidation^38^. The concentration of MDA has been evaluated by using TBARS, which is an indicator of MDA. It is observed from Fig. 6b that the concentration of MDA has significantly increased in cells treated with Ag@ZnO NC as compared to untreated sample with increase in incubation time of up to 20 h. The MDA level increased progressively and is found to be highest at 12 h of incubation (0.010 nmole/μL). However at 24 h incubation, the concentration of MDA is observed to be lesser signifying that MDA might be degraded. However, the concentration of MDA is much less in cells treated with Ag and ZnO NPs (Supplementary Figure 4).

To further elucidate the antifungal mechanism, we tried to investigate the rate of DNA damage in *C. krusei* by using different concentration of Ag NPs, ZnO NPs and Ag@ZnO NC. From Fig. 6c represents the DNA degradation of the fungal cell with Ag@ZnO NC. It can be observed that following 24 h of incubation, there is appearance of a distinct DNA band in the control sample. With 50 μg/mL of NC a slightly less intense band can be observed compared to the untreated sample. However with further increase in concentration of the NC, there is no appearance of DNA band suggesting complete DNA degradation. Ag and ZnO NP treated cells have shown DNA damage in doze dependant manner (Supplementary Figure 4). 500 μg/mL and 1000 μg/mL of the metal and metal oxide NPs have not shown any DNA band suggesting complete DNA degradation. However, Ag@ZnO NC have shown similar results with lower concentration (100 and 250 μg/mL) indicating superior antifungal activity. This might be due to the fact that when fungal cells are exposed to the NC, the later can rupture the cell membrane thereby inducing significant oxidative stress and generating ROS which ultimately lead to DNA degradation. Previous reports on antimicrobial activity of NPs also suggest that oxidative stress in microbial cells could play an important role in inducing DNA damage through generation of ROS^39^. The later attacks DNA thereby producing chain breaks and creating modifications in carbohydrate structures, nitrogen bases by different reactions such as oxidation, nitration, methylation and deamination^40^,^41^.

### Cytotoxicity and Genotoxicity of Ag@ZnO core-shell NC against A431 human epidermoid carcinoma cell line

**Figure 7:**
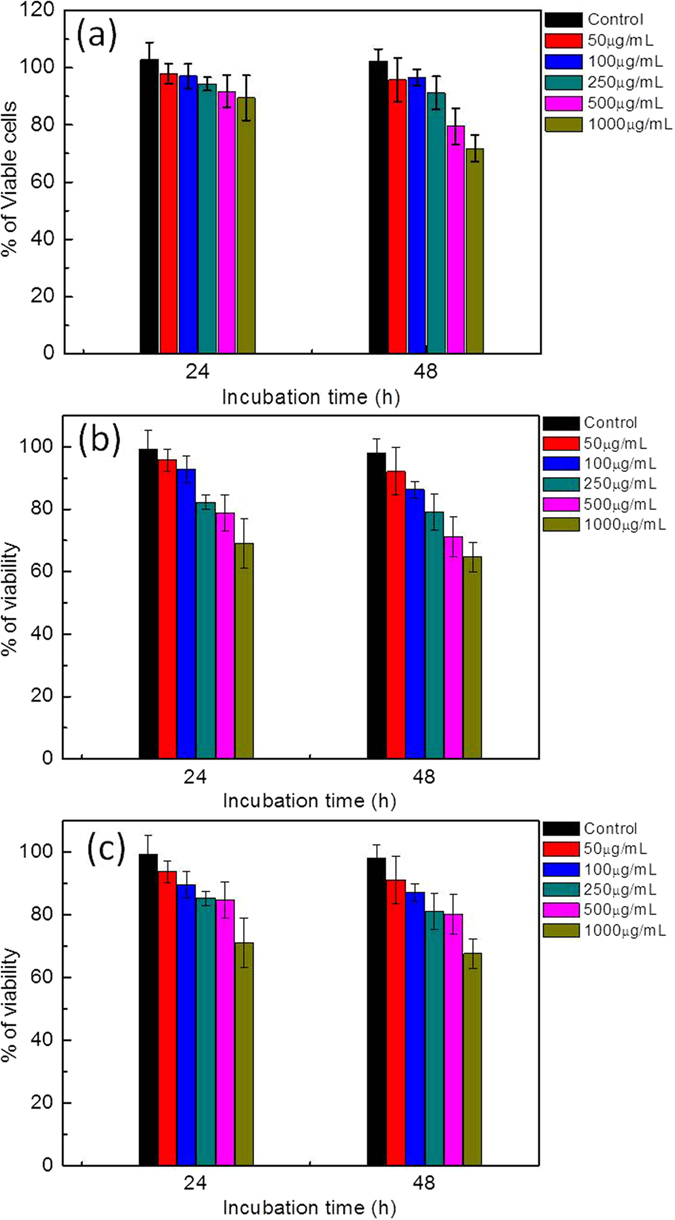
Evaluation of cytotoxicity of (**a**) Ag@ZnO core-shell (**b**) Ag NPs and (**c**) ZnO NPs against A431 cell lines. Cells were incubated with different concentration (50, 100, 250, 500, and 1000 μg/mL) of nanomaterials for 24 and 48 h.

For future application of the as synthesized core-shell NC, it is important to understand their biocompatibility factor. This will ensure that the antimicrobial material will target the pathogenic cells without causing any toxic effect towards the normal cells. Hence, we have investigated the cytotoxicity of the synthesized Ag@ZnO core-shell NC against normal A431 human epidermoid carcinoma cell lines. From Fig. 7(a), it is seen that after 24 h of incubation, as compared to the untreated cells, the percentage of viable cells in the treated samples is very high. Treatment with the optimized concentration of the NC material used in the present study (i.e. 250 μg/mL), has shown 89% cell viability after 48 h of incubation indicating very less toxicity. Under similar experimental condition (250 μg/mL treated for 48 h), Ag and ZnO NPs have shown 79% and 81% cell viability respectively (Fig. 7b,c). Both the NPs have shown very less toxicity towards the cell line used in the study. The reason might be due to the biocompatibility of the as synthesized Ag NPs. However, previous studies with chemically synthesized Ag NPs and ZnO NPs have shown considerable toxicity towards the above mentioned cell line at lower concentrations^42^,^43^. In the present study, least cytoxicity has been observed with Ag@ZnO NC which might be due to the core-shell morphology which may not have allowed the leaching of the silver ion, thereby maintaining its antimicrobial efficiency. Das *et al*. have observed similar results with chemically synthesized Ag@ZnO core-shell NCs^44^.

Since core shell NC are used as topical antifungal agents, the cytotoxicity of the as synthesized Ag@ZnO NC has been observed against the endothelial cell line A570. From the MTT data (Supplementary Figure 6) it has been observed that the above mentioned material has shown very less toxicity towards A570 cell line. The cell viability has been observed to be 91% following 48 h of incubation.

**Figure 8:**
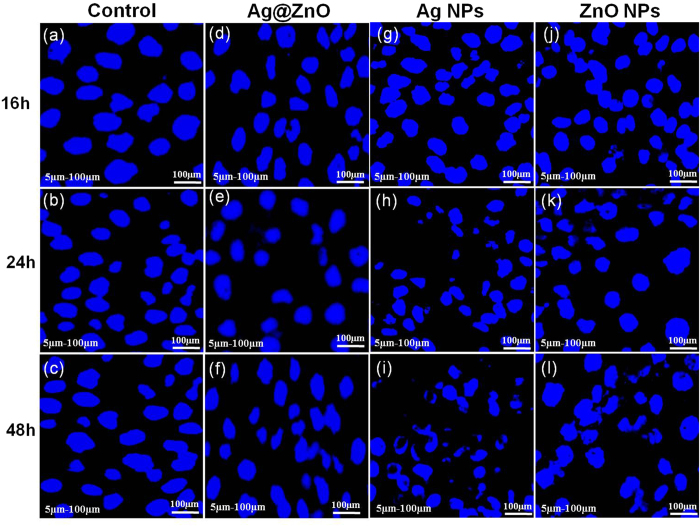
Genotoxicity of nanomaterials against A431 normal cell lines analyzed through DAPI staining. Cells were incubated with 250 μg/mL of nanomaterials (**a–c**) control, (**d–f**) Ag@ZnO NC treated, (**g–i**) Ag NPs treated, (**j–l**) ZnO NPs treated.

Genotoxicity of Ag NPs, ZnO NPs and Ag@ZnO NC against the above mentioned cell line has been evaluated through fluorescence microscopy after staining with DAPI. Figure 8f clearly shows that the nuclear morphology of A431 cells is unaltered in the presence of 250 μg/mL NC up to 48 h of incubation. This confirms that the synthesized NC used in the study does not have any genotoxicity towards the human epidermoid carcinoma cell line. Similar results have been obtained with 250 μg/mL of Ag and ZnO NPs (Fig. 8i,l respectively).

**Figure 9:**
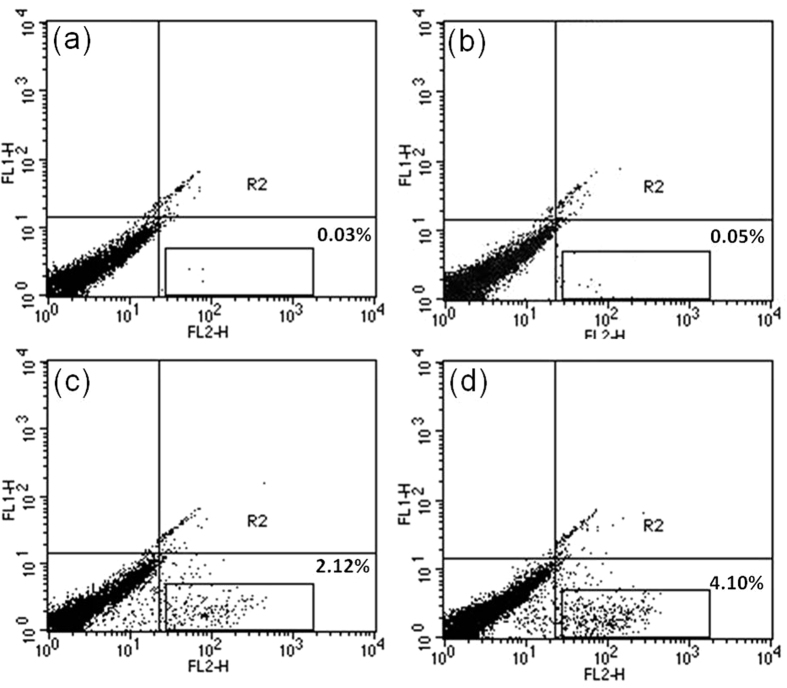
FACS were performed to check cytotoxicity of Ag@ZnO NC against A-431 normal cells line. Incubation time was **(a)** 24 h untreated **(b)** 48 h untreated **(c)** 24 h treatment **(d)** 48 h treatment. Propidium iodide staining assay was used to determine cytotoxicity. Cells were incubated in 250 μg/mL of Ag@ZnO NC.

To confirm the cytotoxicity induced in treated A431 cell lines by incorporation of Ag@ZnO NC, propidium iodide (PI) uptake assay has been performed and quantified through FACS. From the results shown in Fig. 9, it is evident that the population of cells in the lower right quadrants, which is stained by PI, corresponds to the percentage of nonviable cells. It has increased to 2.12% and 4.10% after treatment with 250 μg/mL of Ag@ZnO NC for 24 and 48 h respectively (Fig. 9c,d). However, uptake of PI is very less in control or untreated cells after 24 and 48 h incubation (Fig. 9a,b). Similarly we have also performed the FACS of Ag and ZnO NPs with the above mentioned cell line. The obtained data suggests that the toxicity of Ag NPs and ZnO NPs is marginally higher than Ag@ZnO (Supplementary Figure 5). Considering these results it is expected the as synthesized Ag@ZnO core-shell NCs show effective antibacterial activity against *C. krusei* while it does not offer severe toxicity towards normal A431 cells.

## Conclusion

In summary, antifungal activity of Ag@ZnO NC with core-shell morphology is investigated against opportunistic pathogen *C. krusei*. From experimental observations, present report communicates a promising consequence. Core-shell NC is obtained by coating of zinc oxide on biogenic silver NPs synthesized using *Andrographis paniculata* and *Aloe vera*. Comparative antifungal data indicates that NC material could inhibit the growth of the targeted pathogen at a lower dose size (250 μg/mL) than that of pure Ag and ZnO NPs. Our investigation suggests that the antifungal mechanism of core-shell NC system could be attributed to the generation of ROS which may degrade cellular compounds. Additionally the core-shell NC was found to be less toxic towards normal A431 human epidermoid carcinoma cell lines. These findings justify further studies for possible biomedical application of the proposed Ag@ZnO core-shell NC material.

## Materials and Methods

### Bio-synthesis of Ag NPs

*Andrographis paniculata* (AP) and *Aloe vera* (AV) were collected from the local market of Bhubaneswar. 10 gm of clean and washed leaves of AP and AV each were taken in 100 mL of de-ionized (DI) water. They were macerated thoroughly by motor pestle for 15 minutes and boiled at 60 °C for 30 minutes than filtered using Whatman filter paper 1. The clear filtrate was collected for biosynthesis of NPs. 80 ml of Silver perchlorate hydrate (Sigma-Aldrich, 99%) solution was taken, kept under stirring condition on hot plate. At 90 °C, 10 mL of clear filtrate aqueous solution obtained from the plant extract mixture (10 times diluted) was added under stirring condition. The color of the solution was changed to yellowish-brown which indicated the formation of metallic Ag NPs. Four parameters were monitored for optimizing the process of synthesis i.e. temperature, reaction pH, plant extract dilution factor and concentration of silver salt. pH of the Silver perchlorate hydrate solution was adjusted by adding 1 M NaOH and 0.5 N HCl. The process was first optimised for the reaction pH and temperature. Four different pH (pH 4.0, 7.0, 10.0, and the natural pH = 6.34) of the silver salt was taken and synthesis was observed at four different reaction temperatures (4 °C, 50 °C, 70 °C, 90 °C and room temperature i.e. 28 ± 2 °C). Similarly four different dilutions of the plant extract were made (1×, 10×, 100×, 1000×). Each dilution was used for biosynthesis at four different concentration of silver salt solution (0 mM, 1 mM, 2 mM, 3 mM, and 4 mM). Spectroscopic results were used to obtain the optimized synthesis condition. These two parameters were further used to optimize results for dilution rate and concentration of Ag NPs.

### Synthesis of Ag@ZnO NC

ZnO NPs were coated on the surface of biogenic Ag NPs via colloidal technique. 2 mL of 2 mM sodium hydroxide solution was added to 10 mL of 10 mM zinc nitrate hexahydrate (Sigma Aldrich, 98%) to obtain a white precipitate of zinc hydroxide. The obtained precipitate was dissolved by the addition of excess amount of sodium hydroxide solution to form sodium zincate. Then 1 mL sodium zincate solution was diluted to 5 mL with DI water and added to 100 mL of biologically synthesized Ag NPs at 95 ± 2 °C under vigorous stirring. It was stirred for 30 minutes to prepare Ag@ZnO NC. Change in color from yellowish brown to black indicated the formation of Ag@ZnO core-shell NC.

### Characterization of the materials

Synthesis of Ag NPs and Ag@ZnO NC was followed by UV-visible spectroscopy (Shimadzu UV-1800). Bio-synthesized and purified NPs were characterized by using various instrumental techniques. The morphology of the materials was investigated by transmission electron microscopy (TEM, JEOL-JEM-2010). The crystal structure of the material was investigated by X-ray diffraction technique (D/Max 2005, Rigaku). Fourier Transform infrared spectroscopy (FTIR) (Shimadzu 8201PC, Japan) was used to investigate the presence and nature of surface functional groups.

### Determination of total phenolic contents

The total phenolic content of *Andrographis paniculata*, Aloe vera plant extract as well as Ag@ZnO core shell NCs has been assayed by Folin–Ciocalteau’s method. Being one of the member of phenolic family, galic acid is chosen as a standard for determining the phenolic content. 0.1 ml of *Andrographis paniculata*, Aloe vera plant extract, mixture of both the above mentioned plant extracts (1:1 ratio) and Ag@ZnO samples were taken in conical flasks and final volume was adjusted to 46 ml by adding 45.9 ml distilled water. Then 1 ml Folin- Ciocalteau reactive solution (1 N) was added and incubated at room temperature for 3 min. Finally, 3 ml (2%) sodium carbonate solution was added and mixtures were put on shaker for 2 h at room temperature followed by measurement of OD at 760 nm in spectrophotometer. The phenolic compound content was expressed as gallic acid equivalent (GAE, μg).

### Antifungal assay

Comparative antimicrobial activity of Ag NPs, ZnO NPs and Ag@ZnO NC was investigated against *Candida krusei.* Fungal growth inhibition was performed by inoculating 1 × 10^6^ CFUs of *C. Krusei* cells into 20 mL of PDB with different concentrations (0, 50, 100, 250, 500, 1000 μg/mL) of nanomaterials and incubated in shaking incubator at 30 °C in 200 rpm for 24 h. Then 100 μL of sample was taken at 3 h interval and serially diluted (10^−1^ to 10^−3^) in normal saline solution (0.9% NaCl). Samples were taken from 10^−3^ dilution and plating was done on PDA by drop plate method to determine inhibition of cell growth by CFU assay. The Minimum inhibitory concentration (MIC) for each of the above mentioned nanomateirals was determined by considering the lowest concentration of the materials which could completely inhibit the growth of *C. krusei*.

### Live/dead staining

To check viability of *C. krusei* after treatment with 250 μg/mL of Ag@ZnO NC, live/dead staining was performed by using fluorescein diacetate (FDA) (Sigma-Aldrich) and propidium iodide (PI) (MP Biomedicals). The protocol followed is same as reported by Ehgartner *et al*.^45^. Initially 1 × 10^6^ cells of *C. krusei* were incubated with 250 μg/mL of Ag@ZnO NC for 0, 12 and 24 h. It was then centrifuged to remove culture media and washed with 1x PBS. Then pellets were again resuspended in 1x PBS. Subsequently 10 μg/mL FDA and 20 μg/ml PI was added into untreated and treated samples and incubated in the dark at room temperature for 15 minutes. Cell viability was analyzed under fluorescence microscope (Floid Cell Imaging Station, Life Technology).

### FE-SEM of treated fungal cell

Samples of treated and untreated *C. krusei* cells for Field Emission Scanning Electron Microscope (FE-SEM) were prepared according to previously reported protocol^46^. Initially 1 × 10^6^ cells of *C. Krusei* were treated with 250 μg/mL of Ag@ZnO NC for 12 and 24 h. Then treated and untreated cells were fixed with 4% glutaraldehyde (Sigma-Aldrich) in 0.2 M sodium cacodylate buffer (pH 5.5 to 6.0; Sigma-Aldrich). The cells were washed with cacodylate buffer, and dehydrated using ethanol. The samples (untreated and treated cells) were further sputter coated with gold and analyzed under FE-SEM.

### Determination of reactive oxygen species (ROS)

Generation of intracellular reactive oxygen species (ROS) in *C. krusei* cells were measured qualitatively by using 2′,7′-dichlorofluorescin diacetate (DCF-DA) through flourescence microscope as described by Wojtala *et al*.^47^. DCF-DA is a specific dye for intracellular reactive oxygen species (ROS) which enters into the cells passively and reacts with ROS to form highly fluorescent dichlorofluorescein (DCF). 1 mL sample of Ag NPs, ZnO NPs and Ag@ZnO NC (250 μg/mL) treated cells were taken and washed with 1x PBS by centrifugation at 8,000 rpm for 15 minutes. Then cell pellets were resuspended in 1x PBS buffer and 10 μM of DCF-DA was added and incubated for 10 minutes at 37 °C under dark condition. The images were analysed under fluorescence microscope.

### Determination of lipid peroxidation by malondialdehyde (MDA) assay

MDA is a common end product of lipid peroxidation. Estimation of MDA through its reaction with thiobarbituric acid (TBA) forms a pink colored MDA-TBA complex which predicts the degree of damage in microbial cell membrane^48^. Lipid peroxidation assay kit (Sigma-Aldrich) was used to determine the concentration of MDA in the reaction system.

### DNA degradation

*Candida krusei* cells were treated with different concentrations (0, 50, 100, 250, 500, 1000 μg/mL) of Ag NPs, ZnO NPs and Ag@ZnO for 24 h. DNA was isolated from treated and untreated cells followed by previously reported protocol^49^. The isolated DNA pellets were dissolved in Tris EDTA buffer and 5 μL of the DNA samples were separated on 0.9% agarose gel prepared in Tris acetate EDTA buffer that was stained with ethidium bromide (1 μg/mL). Image was taken in gel documentation system (Gel Doc^TM^ EZ Imager, Bio-Rad).

### Cytotoxicity assay

A431 human epidermoid carcinoma cell lines were obtained from American Type Culture Collection (ATCC), Manassas, USA. Cells were cultured in Dulbecco’s modified Eagle’s medium (DMEM) supplemented with L-glutamine (4 mM), penicillin (100 units/mL), streptomycin (100 μg/mL) and 10% (v/v) fetal bovine serum (growth medium). Firstly, 3 × 10^5^ cells were seeded in 24 well plate and incubated for 48 h at 37 °C in presence of 5% CO_2_ in humidified incubator. Then supernatants were aspirated from the wells and fresh growth medium was added, which was supplemented with desired concentrations of Ag NPs, ZnO NPs and Ag@ZnO NC in the range of 0, 50, 100, 250, 500, 1000 μg/mL. Cells were incubated at 37 °C in humidified incubator in presence of 5% CO_2_ for 24 and 48 h. The cytotoxicty of materials against A431 cell lines was performed followed by standard methylthiazole tetrazolium (MTT) assay^50^ which was quantified through Microplate reader (Biorad, USA, model 680). In addition MTT assay was done with A570 endothelial cell line by taking the above mentioned concentrations of Ag@ZnO NC. The cells were maintained in Ham’s F12 medium.

### Genotoxicity

DAPI (4′,6-Diamidino-2-Phenylindole, Dihydrochloride) staining was performed to evaluate the genotoxicity of Ag NPs, ZnO NPs and Ag@ZnO NC against A431 cell lines. Cells were treated with 250 μg/mL of NC for 16, 24 and 48 h, washed with 1x PBS buffer to remove media and NC. The treated and untreated cells were covered with 300 nM DAPI stain and incubated for 10 minutes in dark at room temperature. Then cells were washed with 1x PBS to remove the stain solution. The nuclear morphology of the cells was analyzed under fluorescence microscope at 40x. Untreated cells were used as control.

### Determination of cytotoxicity by FACS

Uptake of PI by cells was evaluated through FACS according to previously described protocol^51^. A431 cell lines (3 × 10^5^ cells/well) were seeded in 24 well plate and incubated at 37 °C (in the presence of 5% CO_2_) for 48 h. The supernatant was discarded with the help of aspirator and cells were incubated with 250 μg/mL of Ag NPs, ZnO NPs and Ag@ZnO NC (suspended in culture medium) for 24 and 48 h. Treated and untreated cells were fixed using 70% ethanol at 4 °C for 1 h. Then cells were resuspended in 1x PBS and 50 μg/mL of PI was added to each wells and incubated at 37 °C for 15 minutes. Samples were analyzed using a FACS calibur flow cytometer and CELL-Quest software (Becton-Dickinson, BD Biosciences, Franklin Lakes, NJ). In total, 10000 cells were analyzed per sample.

## Additional Information

**How to cite this article**: Das, B. *et al*. Understanding the Antifungal Mechanism of Ag@ZnO Core-shell Nanocomposites against *Candida krusei*. *Sci. Rep.* **6**, 36403; doi: 10.1038/srep36403 (2016).

**Publisher’s note:** Springer Nature remains neutral with regard to jurisdictional claims in published maps and institutional affiliations.

## Supplementary Material

Supplementary Information
