# Supplementary material for: Understanding the Antifungal Mechanism of Ag@ZnO Core-shell Nanocomposites against Candida krusei
Source: Sci Rep. 2016 Nov 4;6:36403. doi: 10.1038/srep36403 (PMC5095647; doi:10.1038/srep36403)
Supplement: Supplementary Information [file srep36403-s1.pdf]

Supporting Data

Understanding the Antifungal Mechanism of Ag@ZnO Core-shell Nanocomposites against *Candida krusei*

Bhaskar Das, Md. Imran Khan, R. Jayabalan, Susanta K. Behera, Soon-Il Yun, Suraj K. Tripathy, Amrita Mishra

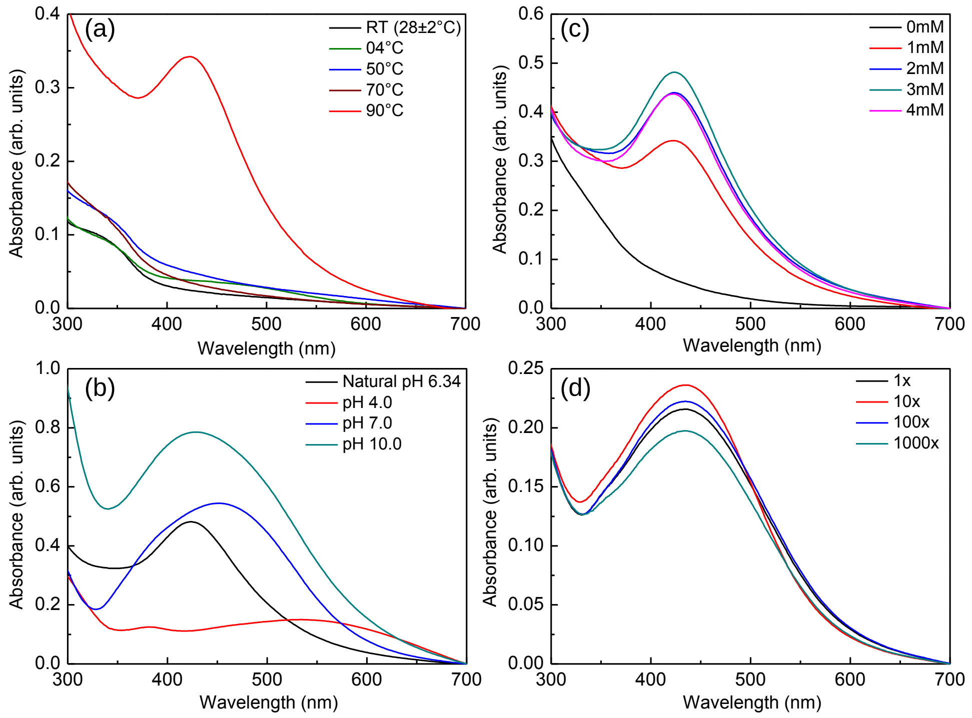

Supplementary Figure 1: Biosynthesis of silver nanoparticles (NPs) was optimized **(a)** at different temperature **(b)** At different pH **(c)** by using different concentration of silver salt (AgClO4) **(d)** by using different concentration of leave extracts

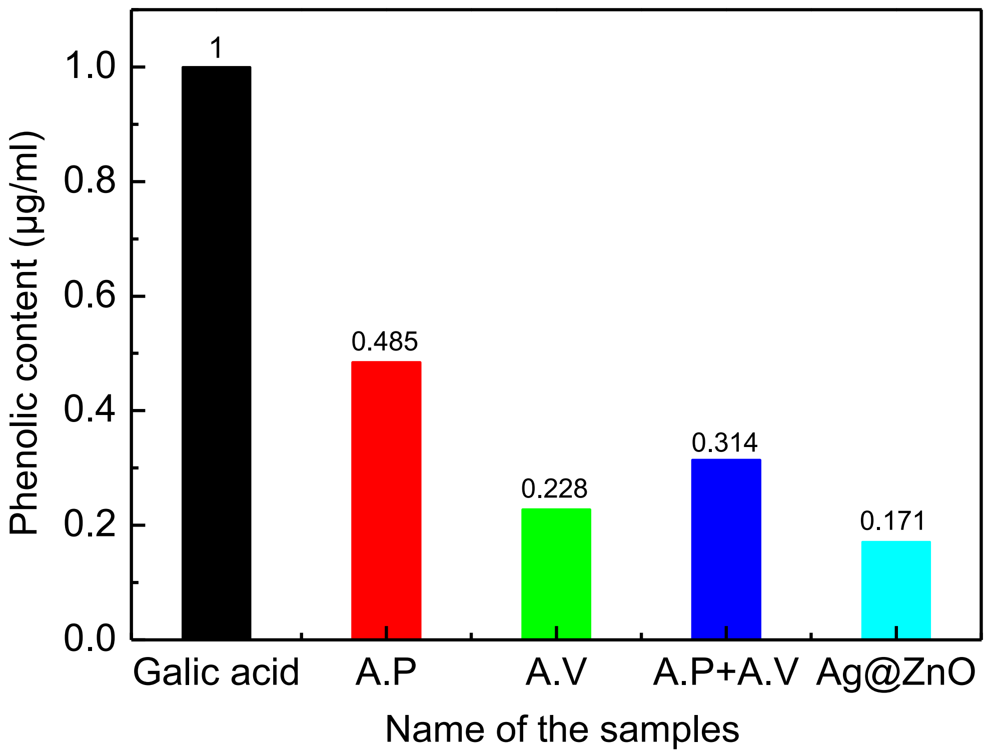

Supplementary Figure 2: Determination of total phenolic contents.

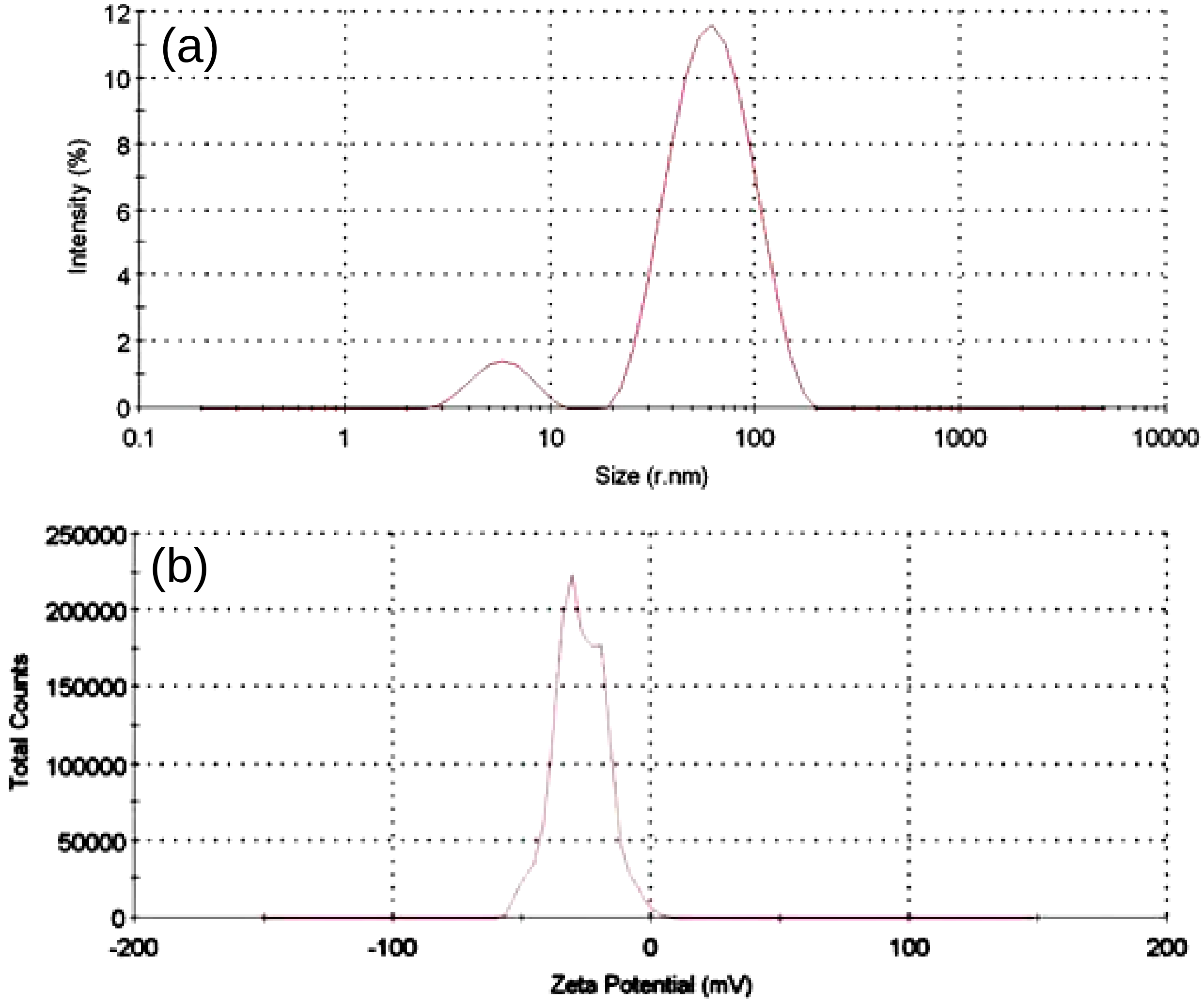

Supplementary Figure 3: Ag@ZnO characterized by Dynamic Light Scattering (DLS) **(a)** Size distribution **(b)** zeta potential

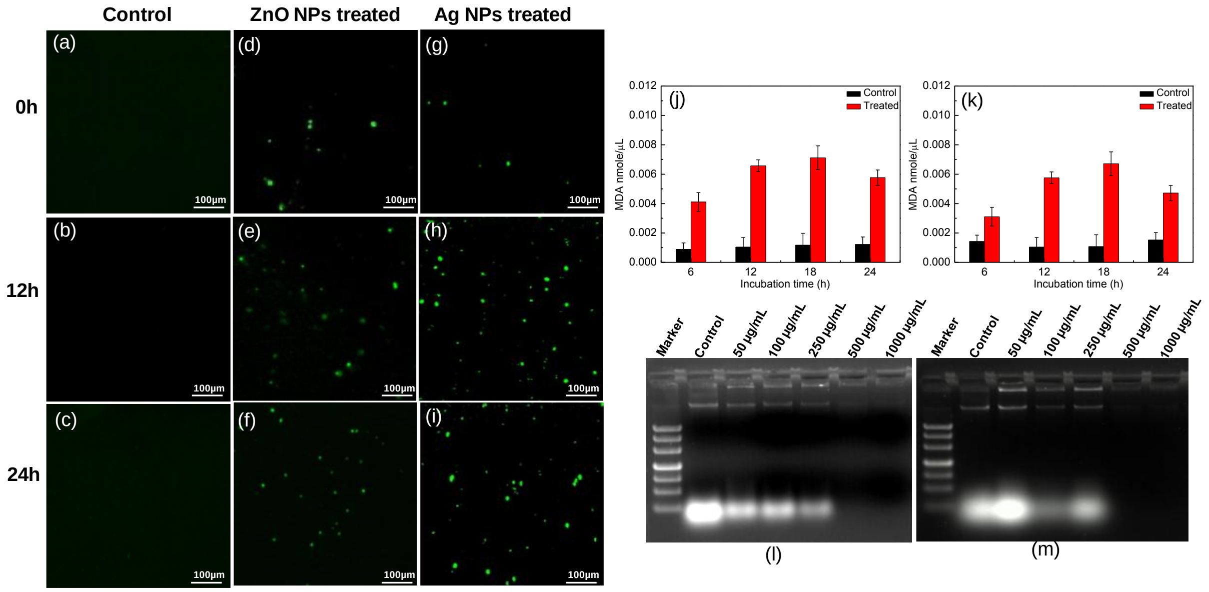

Supplementary Figure 4: Determination of ROS **(a, b and c)** Untreated **(d, e and f)** AgNPs treated **(g, h and i)** ZnO NPs treated. Lipid peroxidarion assay **(j)** Ag NPs treated **(k)** ZnO NPs treated. DNA Degradation assay **(l)** Ag NPs treated **(m)** ZnO NPs treated

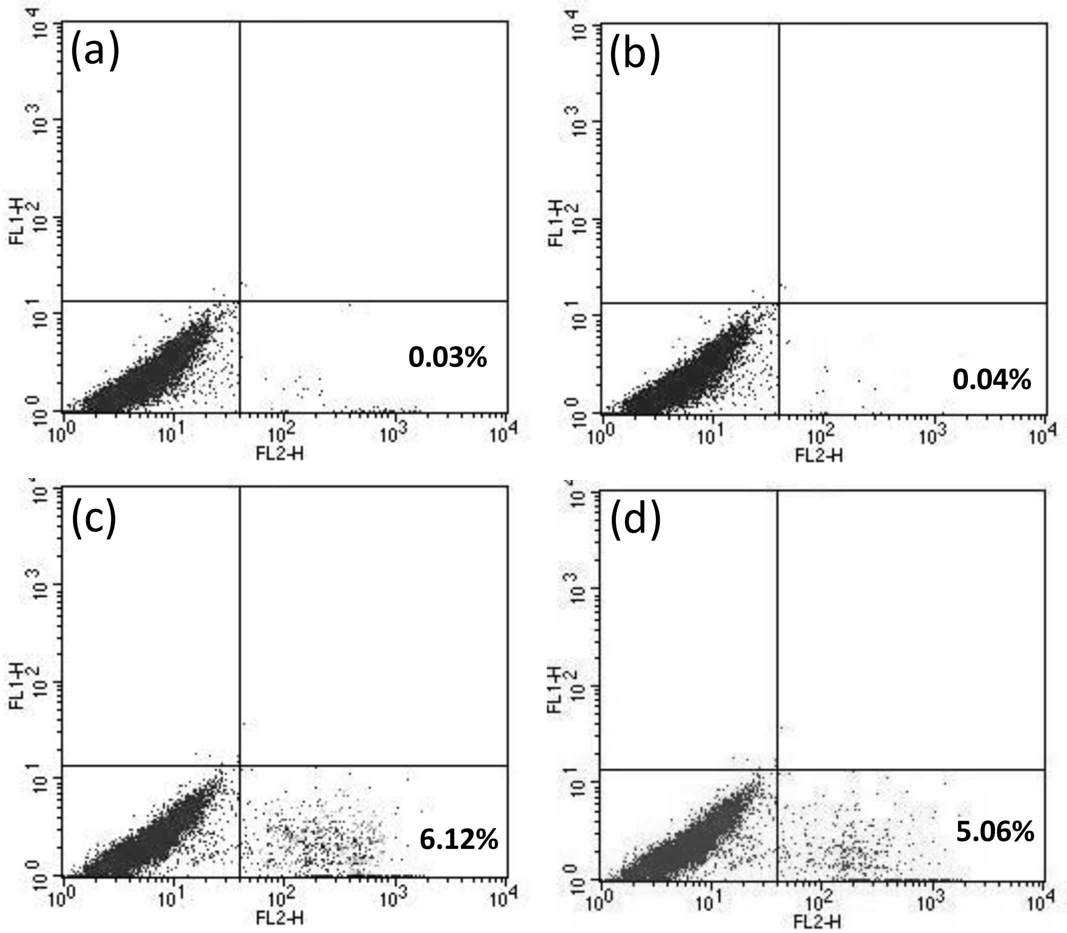

Supplementary Figure 5: Cytotoxicity of Ag and ZnO NPs against A431 normal cell lines determined through FACS (a and b) control (c) Ag NPs treated (d) ZnO NPs treated. Propidium iodide staining assay was used to determine cytotoxicity. Cells were incubated in 250µg/mL of Ag and ZnO naomaterials for 48h.

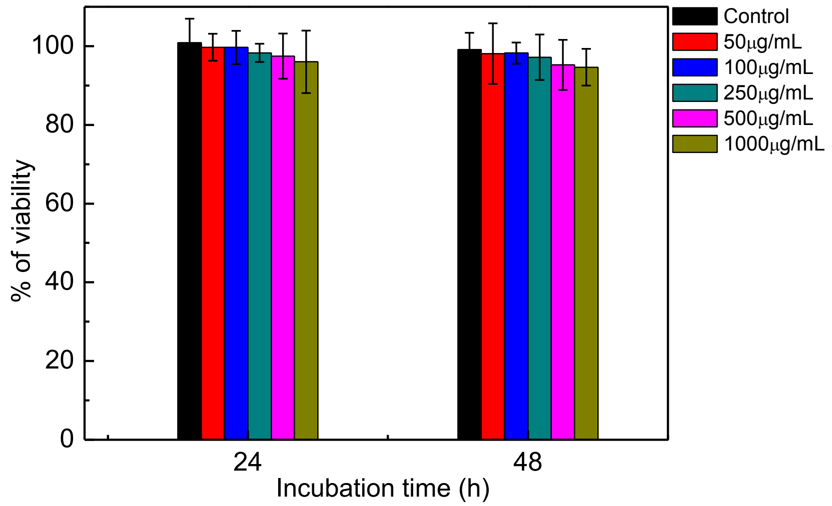

Supplementary Figure 6: Evaluation of cytotoxicity of Ag@ZnO core-shell against A570 cell lines

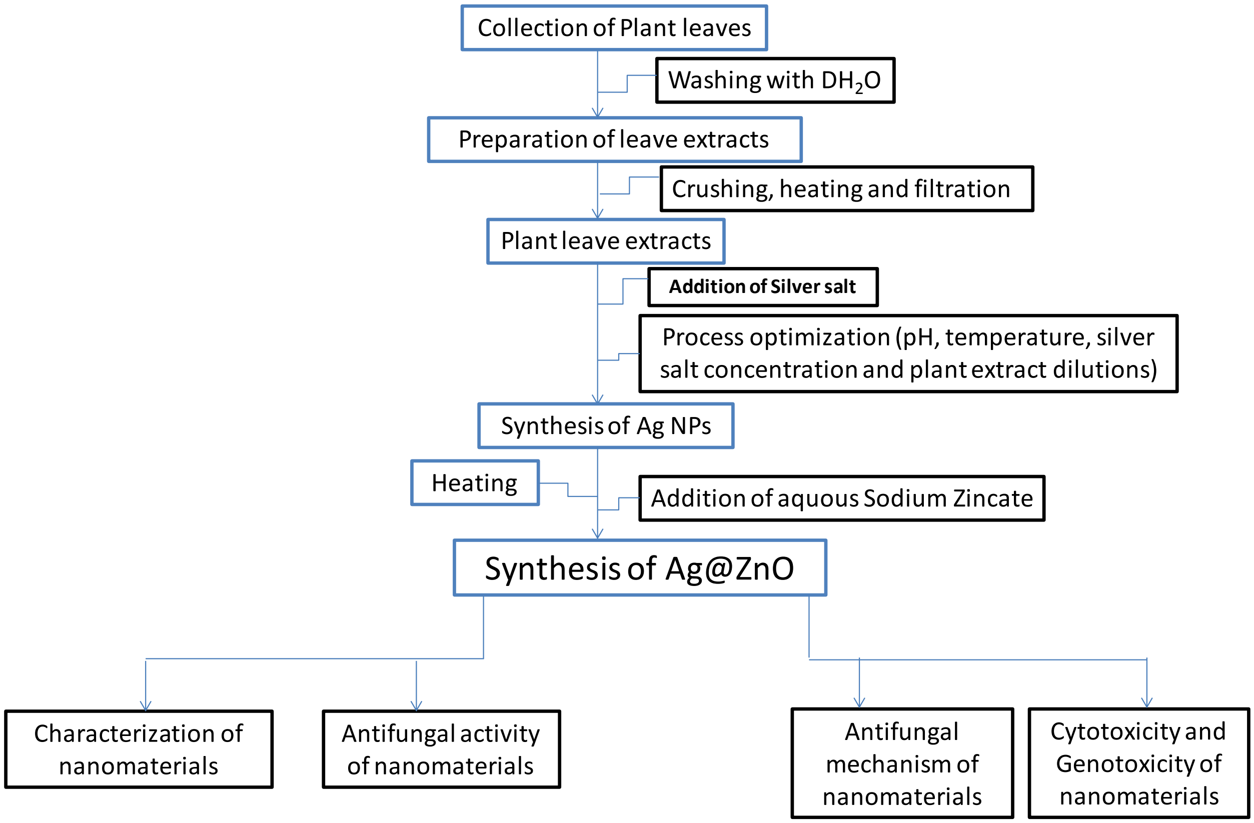

Supplementary Figure 7: Flow chart of experiment

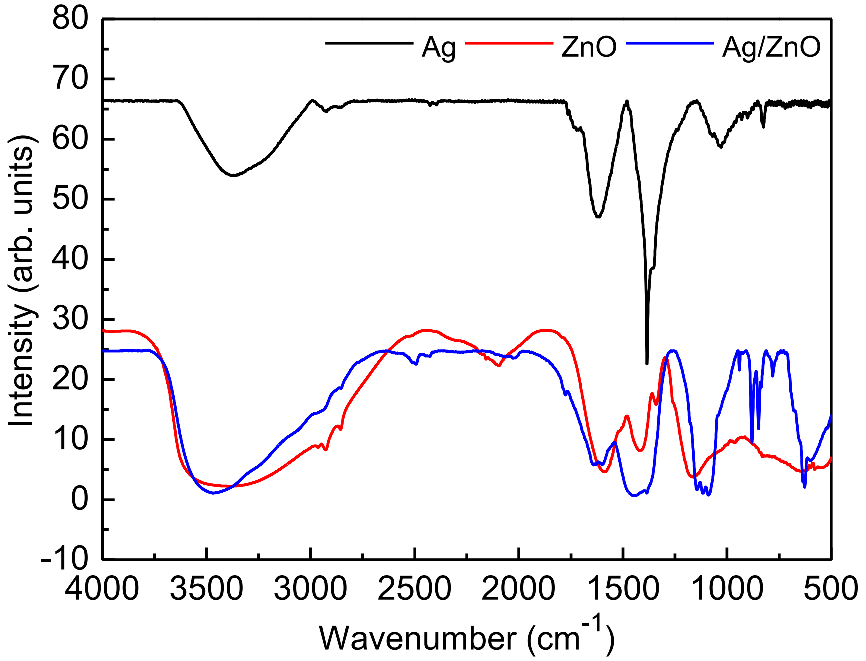

Supplementary Figure 8: FTIR spectra of Ag, ZnO, and Ag@ZnO

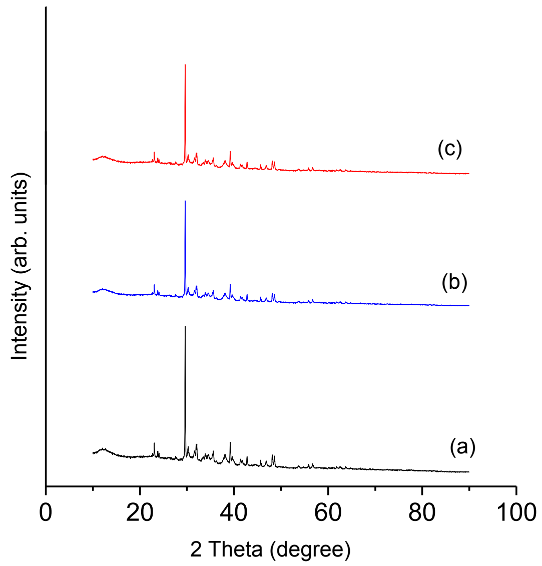

Supplementary Figure 9: XRD pattern of Ag@ZnO NC, (a) without treatment, (b) treated with PBS, and (c) treated with fungus growth culture media
